# Functional Interplay between RNA Viruses and Non-Coding RNA in Mammals

**DOI:** 10.3390/ncrna5010007

**Published:** 2019-01-14

**Authors:** Nkerorema Djodji Damas, Nicolas Fossat, Troels K. H. Scheel

**Affiliations:** 1 Copenhagen Hepatitis C Program (CO-HEP), Department of Immunology and Microbiology, Faculty of Health and Medical Sciences, University of Copenhagen, DK-2200 Copenhagen, Denmark; ndamas@sund.ku.dk (N.D.D.); nfossat@sund.ku.dk (N.F.); 2 Department of Infectious Diseases, Hvidovre Hospital, DK-2650 Hvidovre, Denmark; 3 Laboratory of Virology and Infectious Disease, Center for the Study of Hepatitis C, The Rockefeller University, New York, NY 10065, USA

**Keywords:** virus, RNA, micro-RNA, long non-coding RNA, sub-genomic flavivirus RNA, RNA interference, post-transcriptional regulation, RNA interactions

## Abstract

Exploring virus–host interactions is key to understand mechanisms regulating the viral replicative cycle and any pathological outcomes associated with infection. Whereas interactions at the protein level are well explored, RNA interactions are less so. Novel sequencing methodologies have helped uncover the importance of RNA–protein and RNA–RNA interactions during infection. In addition to messenger RNAs (mRNAs), mammalian cells express a great number of regulatory non-coding RNAs, some of which are crucial for regulation of the immune system whereas others are utilized by viruses. It is thus becoming increasingly clear that RNA interactions play important roles for both sides in the arms race between virus and host. With the emerging field of RNA therapeutics, such interactions are promising antiviral targets. In this review, we discuss direct and indirect RNA interactions occurring between RNA viruses or retroviruses and host non-coding transcripts upon infection. In addition, we review RNA virus derived non-coding RNAs affecting immunological and metabolic pathways of the host cell typically to provide an advantage to the virus. The relatively few known examples of virus–host RNA interactions suggest that many more await discovery.

## 1. Introduction

Viruses are likely the most abundant biological entity and the known global virosphere is greatly expanding [1,2]. Viral infections cause significant morbidity and mortality worldwide. Socio-economic and environmental changes increase the frequency and impact of outbreaks, as recently seen for Zika virus (ZIKV), chikungunya virus (CHIKV), Ebola virus (EBOV), and severe acute respiratory syndrome coronavirus (SARS-CoV) [3,4,5]. As obligate intracellular pathogens, viruses make extensive use of host cell machinery to replicate. The interfaces between host and virus have provided extraordinary insights to molecular mechanisms of pathogenesis and yielded valuable therapeutic targets. Viruses further constitute a unique opportunity to advance our understanding of mechanisms of cell biology. Virology already provided numerous tools and basic discoveries in cellular biology [6]. The simple genetic architecture, high diversity, and fast evolution of viruses have made them perfect for subtractive and comparative studies [7,8,9]. Most systematic studies of virus–host interactions have focused on proteins whereas RNA interactions are a more recent focus area.

### 1.1. RNA Viruses

Viruses are classified into two main groups depending on their genetic material. DNA viruses have double- (dsDNA; e.g., *Herpesviridae*) or single-stranded (ssDNA; e.g., *Parvoviridae*) genomes; RNA viruses have double-stranded (dsRNA; e.g., *Reoviridae*), or single-stranded positive sense ([+]ssRNA; e.g., *Flaviviridae*) or negative sense ([−]ssRNA; e.g., *Orthomyxoviridae*) genomes, where orientation is defined for coding mRNA sequence to be of positive polarity. In addition, *Hepadnaviridae* are DNA viruses with RNA intermediates, and *Retroviridae* are (+)RNA viruses with DNA intermediates [10]. RNA viruses have particular high mutation rates due to lack of proof-reading activity and high frequency of recombination events. This allows RNA viruses to rapidly adapt to new environments and undergo intra-host evolution to avoid cellular immune responses or antiviral therapies, conferring RNA viruses a great intrinsic epidemic potential [5,11,12,13,14,15,16]. They therefore also have potential to rapidly select for advantageous interactions with host factors, including cellular RNA, or avoid those that are non-advantageous. With their on average smaller genome size compared to DNA viruses they on the other hand have fewer options for encoding viral non-coding RNA (ncRNA) species.

Viruses strictly depend on the cellular metabolic and translational machinery to accomplish genome replication and assembly of infectious particles. During the whole viral life-cycle, viruses therefore interact with a great number of cellular factors. In addition, some RNA viruses remodel intracellular membranes in order to create protective replication compartments, modulate lipid metabolism, and evade the innate immune response (reviewed in [17,18,19]). The latter is achieved by encoding viral proteins that subvert, interfere with, or degrade innate immune system components. These processes may in addition be mediated through cellular or viral RNA. Thorough understanding of virus–host interactions is therefore critical for prevention and control of viral infections. In this review, we focus on the interplay between RNA viruses and vertebrate RNA upon infection, with special interest for the cellular non-coding transcriptome.

### 1.2. Mammalian Non-Coding Transcriptome

Protein-coding messenger RNAs (mRNAs) are highly conserved even between phylogenetically distant organisms [20,21]. A deeper understanding of genomics has instead revealed a correlation between organism complexity and expression of intra- and intergenic ncRNA [22]. Complete sequencing of the mammalian transcriptome has revealed how more than 80% of the genome is pervasively transcribed, with only 1–2% of transcripts possessing protein-coding potential. This produces a great variety of RNA transcripts with different regulatory potential [23,24,25]. An increasing number of ncRNA species have been characterized and divided into functional classes (reviewed in [26,27]): ribosomal RNAs (rRNAs) regulating protein synthesis and ribosome architecture; transfer-RNAs (tRNAs) acting as adaptor molecules during protein synthesis; small nuclear RNAs (snRNAs) being the central core of the spliceosome machinery; small nucleolar RNAs (snoRNAs) regulating the processing and maturation of rRNAs, tRNAs, snRNAs, and ribosome assembly; micro-RNAs (miRNAs) and piwi interacting RNAs (piRNAs) regulating post-transcriptional gene expression and stability of mRNAs and transposons respectively; ribozymes catalyzing post-transcriptional cleavage reactions; riboswitches that are ncRNA elements binding small molecule metabolites to regulate gene expression in a stereospecific fashion; and finally long non-coding RNAs (lncRNAs) titrating miRNAs and RNA binding proteins and acting as scaffolds for protein and DNA binding to regulate chromatin architecture, epigenetic modifications, and maintaining genome stability. Thus, there are plenty of cellular RNA species that could play important roles during infection. In addition, it is not surprising that viruses evolved to utilize or directly or indirectly alter the function of host regulatory ncRNAs.

### 1.3. Virus–Host Interactions at the RNA Level

Although most of our knowledge of the virus–host interface is at the protein level, several findings highlight a central role for cellular regulatory RNA during viral infection. It has become clear that regulatory RNAs, such as miRNAs and lncRNAs are important regulators of the cellular antiviral response. Conversely, viruses have found ways of utilizing cellular ncRNA. Examples include the use of canonical post-transcriptional miRNA repression to avoid replication in tissue compartments where the virus would risk eliciting immune responses, circumvention of the canonical miRNA pathways to directly use miRNAs for viral RNA accumulation, and the use of tRNAs as primers in retroviral genome replication. Furthermore, RNA virus derived ncRNA, including sub-genomic flavivirus RNAs (sfRNAs), can regulate the host cell upon infection.

With the only recent establishment of high-throughput methods to study RNA interactions, our knowledge on virus–host interactions at the RNA level is predicted to accelerate. The intersecting fields of virology and RNA biology hence could yield many important basic discoveries. Combined with ongoing developments of RNA-based therapy [28], this further holds great promise for future antiviral strategies. Our current knowledge on virus–host interactions at the RNA level is discussed in detail below.

## 2. Direct Interactions between Viral RNA and Host RNAs

### 2.1. micro-RNAs

miRNAs are ~22 nucleotide small regulatory RNAs and post-transcriptional regulators of gene expression. The miRNA genes are transcribed into long pri-miRNAs, which are initially processed in the nucleus to ~70 nucleotide precursor transcripts (pre-miRNAs), by the RNAse III containing microprocessor complex consisting of Drosha and DGCR8. After Exportin-5 mediated export of the pre-miRNA hairpins to the cytoplasm, Dicer-dependent processing produces mature miRNAs [29]. From each pre-miRNA hairpin, a 5′ and a 3′ mature miRNA, given the name extensions -5p and -3p, can be produced. The relative abundance between the two forms varies greatly between miRNAs, tissue types and conditions. Once loaded onto the Argonaute (AGO) protein, the miRNA guides the RNA induced silencing complex (RISC), through a base-pairing mechanism, onto complementary sequences of the target RNA. Nucleotides 2–7 of the miRNA constitutes the core seed region, perfect base pairing to which is typically required for functional interactions. Binding is enhanced by pairing to nucleotide 8, by auxiliary pairing to the region around nucleotide 13–16, and by an adenosine opposite to nucleotide 1, which stabilizes AGO loading. Metazoan miRNAs generally target the mRNA 3′ untranslated regions (UTR), where miRNA seed site interactions induce mRNA translational repression and degradation, depending on the number of available binding sites and degree of base pairing [29].

Mammalian genomes carry thousands of miRNA genes, however different tissue types are characterized by specific expression signatures. Thus, only a subset of miRNA genes is present at a biologically meaningful abundance in any given cell type. In studies of miRNA regulation this is particularly important to consider. The plethora of combinations between miRNA expression profiles and seed site combinations allow for specific fine tuning of gene expression in any tissue or condition. It is estimated that over one third of the overall protein coding gene expression in mammals is fine-tuned by miRNAs [29]. Loss-of-function studies proved that disrupting miRNA gene loci can induce severe developmental defects [30], and expression deregulation in somatic cells has been associated to onset of a broad range of pathological conditions [29]. For example, deletion of the polycistronic miR-17~92 cluster is responsive for severe skeletal defects in humans and mice [31]; mutation of the seed region of human miR-96 has been associated with progressive hearing loss [32], and miR-208 deletion leads to cardiac function decline due to onset of severe structural abnormalities in mice [33]. Disrupting the expression of genes involved in the miRNA biogenesis, such as Drosha and Dicer, as well as impairing the catalytic activity of AGO2 in embryonic cells resulted in lethal phenotypes [34,35]. In somatic cells, de-regulation of the miRNA biogenic pathway affects cell proliferation and viability driving the onset of pathological conditions such as autoimmune responses, organ failure, and cancer [36,37,38].

DNA viruses, for example *Herpesviridae*, are known to exploit host miRNA activity and also encode their own set of viral ncRNAs. The viral non-coding transcriptome targets cellular pathways regulating infection and can control, e.g., the transition between replication and latency [39]. The interplay between DNA viruses and ncRNA is discussed in detail elsewhere [40]. During the last decade, studies have also demonstrated the ability of RNA viruses to directly bind host miRNAs (Table 1) or indirectly manipulate their expression, which is discussed in detail below.

#### 2.1.1. Canonical Repressive Regulation by Host miRNAs

**Figure 1:**
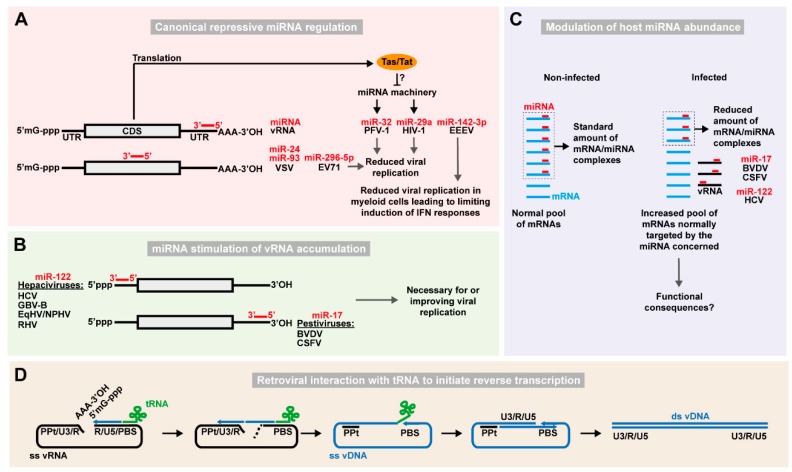
Function of host ncRNAs directly interacting with viral RNA. (**A**) Examples of repressive regulation of RNA virus replication via canonical miRNA-dependent targeting. Host miRNAs (red bars) targeting viral RNA (vRNA) CDS or 3′ UTR are shown. Capped 5′-end (5′mG-ppp) and poly-adenylated 3′ end (AAA-3′OH) of the vRNA are also depicted. Viral proteins such as PFV-1 Tas or HIV-1 Tat may counteract the miRNA machinery. (**B**) Stimulation of RNA virus replication via miRNA-dependent targeting. Hepaciviruses and pestiviruses interact with miR-122 on the 5′ UTR and let-7 and miR-17 on the 3′ UTR, respectively, to stimulate IRES-driven translation, protection from degradation and replication leading to viral RNA accumulation. (**C**) Sponging of host miRNAs by viral RNA affects the expression of cellular mRNAs. In non-infected cells, a defined pool of miRNAs (red bars) targets the mRNA pool (light blue) for inhibition of translation or degradation. In infected cells, vRNA (black) can sponge a significant proportion of specific miRNA species resulting in a reduced available pool of that particular miRNA, and therefore a de-repression of normally targeted mRNAs. These effects have been described for the miRNA/vRNA combinations indicated. (**D**) Retroviral interaction with tRNAs to initiate reverse transcription of the viral RNA. In the *Retroviridae* family, a cellular tRNA (green) complementary to the primer binding site (PBS) sequence in the 5′ region of the viral (+)ssRNA (black) is used to initiate reverse transcription. The complementarity between the long terminal repeat sequences (including the unique 3′ [U3], repeat [R], and unique 5′ [U5] regions) at the ends of the vRNA assists in conversion of the viral genome into ssDNA (blue). Finally, synthesis of the second DNA strand is initiated from the polypurine tract (PPt) region that is resistant to RNAse H, degrading the remaining vRNA, to thereby generate the double-stranded vDNA (ds vDNA) that will get inserted into the cellular chromosome as a provirus.

Many RNA viruses have a rapid course of infection and do not require the complex gene regulation employed by some DNA viruses. Furthermore, due to their rapid evolution, RNA viruses can generally quickly escape disadvantageous canonical miRNA targeting [62]. Compared to negative-strand RNA viruses, positive-strand viruses may have a further advantage through extensive use of RNA recombination [63]. Thus, canonical mRNA-like miRNA targeting is only expected if (i) it is an advantage for the virus to be repressed e.g., under certain circumstances, or (ii) other sequence constraints do not permit the virus to avoid targeting. Canonical miRNA repression of RNA viruses will be discussed below (Figure 1A), excluding studies solely relying on experimental conditions of non-physiological miRNA overexpression.

Enterovirus 71 (EV71) is a (+)ssRNA virus belonging to the *Picornaviridae* that can cause hand, foot, and mouth disease, encephalitis, and neurological complications. Several mammalian cell lines upregulate the expression of miR-296-5p in response to EV71 infection [41]. Two miR-296-5p binding sites were identified in the coding region of the EV71 genome and transfecting cells with synthetic oligos mimicking miR-296-5p activity decreased EV71 replication, whereas inhibition of the endogenous miR-296-5p with antisense oligos promoted viral replication [41]. Mutating the miR-296-5p seed sites abrogated miRNA-dependent inhibition of viral replication, whereas restoration of the seed site in non-targeted isolates rescued the phenotype. However, no clear correlation between miR-296-5p expression and EV71 tissue tropism was identified and none of the miR-296-5p seed sites are conserved across EV71 isolates suggesting either that this may be a less important general mechanism or that other isolates would be targeted elsewhere by the same or other miRNAs. 

Replication of primate foamy virus type 1 (PFV-1) of the *Retroviridae* family is restricted by the expression of cellular miR-32 [42]. Bioinformatic analysis of the PFV-1 3′ UTR identified a putative binding site for miR-32. Both direct and indirect inhibition of miR-32 resulted in accumulation of PFV-1 viral RNA in cell culture, and so did mutagenesis of the miR-32 seed site [42]. In addition, the PFV-1 expressed Tas protein appears to be able to directly interfere with the cellular miRNA machinery and helps PFV-1 to evade the host response [42,64]. Studies also suggested that human immunodeficiency virus 1 (HIV-1) can be directly repressed by the miRNA machinery, with miR-29 binding to the viral 3′ UTR in human T lymphocytes [43]. In this study, virus production was downregulated by exogenous levels of miR-29 for the wild-type but not for a seed site mutant, which however was affected by the corresponding mutant miRNA. The miR-29 site is relatively conserved but not for all HIV-1 isolates. Although cross-linking immunoprecipitation (CLIP) of the AGO protein did identify the miR-29 interaction [65], that and another study [66] did not observe functional effects of the binding. It is possible that miR-29 inhibition may be relevant only in a cell-specific context with high miR-29 levels or low levels of HIV. Anyhow, further studies are needed to determine the biological importance of this interaction. 

Depletion of miRNA-pathway components in human somatic cells with an intact innate immune system could help resolve this issue and allow to further study any miRNA mediated effect on viral infection. In this regard, one study showed that the deletion of Dicer did not increase the infection kinetic of HIV-1, dengue (DENV), West Nile (WNV), yellow fever (YFV), Sindbis (SINV), Venezuelan equine encephalitis (VEEV), measles (MV), influenza A (IAV), vesicular stomatitis (VSV), or reovirus (ReoV), suggesting that these viruses are largely not subject to miRNA regulation [67]. Contrarily, DENV was slightly stimulated by the presence of Dicer. Similarly, another study used a leaky gene-trap allele of *DICER1* to overcome the lethality generally afflicting *DICER1* knock-out embryos, and obtained adult mutant mice depleted of Dicer but with no apparent defect otherwise [44]. Taking advantage of this model, different DNA and RNA viruses were screened, and it was observed that infection by VSV, a (−)ssRNA virus of the *Rhabdoviridae* family, was enhanced in absence of Dicer. Two cellular miRNAs, miR-24 and miR-93, would recognize binding sites on the viral genome and their expression decreased VSV titers. Indeed, the involvement of these two miRNAs in attenuating VSV infection was further confirmed by mutating their binding sites on the viral genome, which increased the susceptibility of wild type mice to VSV infection.

The examples described above suggest that miRNAs play a role in the cellular response to prevent viral infection. While that seems like a winning situation for the host cell, this mechanism may also be exploited by the virus. For example, in the case of HIV, the inhibition by miR-29 was suggested to favor and maintain the latency phase. Eastern equine encephalitis virus (EEEV) is a highly virulent mosquito-borne (+)ssRNA virus of the *Togaviridae* family causing systemic disease or encephalitis with high mortality. EEEV has impaired replication in myeloid cells and avoids the activation of type I interferon (IFN) responses [68]. Combining bioinformatic predictions and luciferase reporter constructs, several binding sites for the hematopoietic-specific miR-142-3p were identified in the EEEV 3′ UTR [45]. Interestingly, EEEV does not replicate in human macrophages and dendritic cells but does so in the latter isolated from miR-142-knock-out mice. Moreover, EEEV mutants carrying deletions of the miR-142-3p sites had increased replication in myeloid cells, whereas trans-complementation of miR-142-3p in other cell types attenuated wild-type infection. In line with this, mutant viruses, unlike the wild-type, elicited type I IFN responses and were attenuated in wild-type mice. The EEEV wild-type and miR-142-3p seed site mutants, however, had similar infection kinetics in IFN-α receptor knock-out mice. These data suggested that EEEV specifically restricts its own replication in myeloid cells to avoid induction of IFN responses [45]. Interestingly, the virus apparently at the same time depends on the miR-142-3p seed containing sequence in mosquitos, which could be a way to preserve this mechanism. The mechanism appears to be EEEV specific and is not conserved for the related VEEV. This exemplifies tropism-specific mechanisms that RNA viruses can adopt to use antiviral properties of miRNAs in ways that can influence pathology. This concept is also being explored in vaccination approaches using modified viruses attenuated in specific tissues through insertion of miRNA sites. This field is reviewed by Fay and Langlois in this issue [69]. Given the tissue-specific expression of miRNAs and that infection studies often focus on one tissue type, other cases similar to that of EEEV may await discovery. Thus, although cases of canonical miRNA activity acting antiviral clearly exist, RNA viruses may also utilize canonical miRNA activity to their own advantage.

#### 2.1.2. Stimulation of Viral RNA Accumulation by Host miRNAs

In addition to being regulated by canonical miRNA activity, viruses can also redirect miRNAs to directly promote their replication (Figure 1B). A milestone in the appreciation of virus-miRNA interactions was reached with the study of hepatitis C virus (HCV) infection. HCV is a (+)ssRNA virus of the *Flaviviridae* family causing chronic infection of the human liver leading to fibrosis, cirrhosis, and hepatocellular carcinoma. In ground-breaking work published in 2005, it was shown that HCV replication is critically dependent on the abundant liver-specific miR-122 and that miR-122 binds two sites located in the HCV 5′ UTR [46,47]. This was surprising for two reasons, given that miRNAs normally are negative regulators and that they typically exert their action at the 3′ UTR. Mutating the binding sites in the viral genome, as well as directly blocking miR-122 with antisense oligonucleotides demonstrated that this non-canonical interaction is required to sustain HCV replication [48]. Structural dissection of the miR-122/HCV 5′ UTR interaction revealed that the recruitment of the AGO protein in proximity of the internal ribosome entry site (IRES) promotes translation, stabilizes the IRES architecture and protects the 5′ end of the un-capped viral genome from exonuclease activity and thereby RNA decay [49,50,51,52,53,54,55]. It was further suggested that miR-122 acts as a switch between translation and replication by competing with binding of poly(rC)-binding protein 2 (PCBP2) to the same region [56].

Until recently, studying whether viral stimulation by miR-122 is evolutionarily conserved has been challenging, given that GB virus B (GBV-B), a virus of unknown origin infecting small New World monkeys, was the only known HCV-relative in the *Hepacivirus* genus [70]. Using so-called replicon systems, allowing replication but not virus production, miR-122 was found to also stimulate GBV-B by binding to its 5′ UTR. For GBV-B, the presence of miR-122 was not essential for replication, however [71]. With the introduction of deep sequencing techniques, the search for HCV-related hepaciviruses during the recent years finally identified a number of such viruses in horses, rodents, monkeys, bats, and cows and some of these may provide long sought-after animal models for the study of HCV [72,73,74]. Interestingly, the presence of one or two miR-122 binding sites is conserved among most of these different viruses. Studies of the equine non-primate hepacivirus (NPHV/EqHV), showed that miR-122 binds the viral genome in vivo and that the interaction with the 5′ end of the viral genome also promotes translation [75,76,77]. Rodent hepacivirus (RHV) isolated from Norway rats is of particular interest as a small animal model for HCV [78,79]. Interestingly, unlike in wild-type mice this virus fails to replicate in miR-122 knock-out mice [78]. Thus, miR-122 dependency appears to be a general phenomenon among hepaciviruses. Since miR-122 independent hepaciviruses with no attenuation compared to wild-type can be generated in the laboratory, it is interesting to speculate whether hepaciviruses prefer miR-122 dependency in order to benefit from the tolerogenic environment of the liver [76].

The HCV/miR-122 interaction has emerged as an interesting clinical target. As a first-in-class, development of locked nucleic acid (LNA) based antisense oligos inhibiting miR-122 led to inhibition of the infection [80]. Significant viral load reduction after inhibitor therapy was demonstrated first in HCV infected chimpanzees and subsequently proved efficacious in phase II clinical trials. Improved pharmacokinetics even led to one-shot virologic cure for few patients with chronic HCV infection; a condition which just few years ago was incurable in most patients [81,82,83]. Even if this therapy may not reach the market, its success in clinical trials certainly encourages similar approaches for other diseases. Furthermore, the employment of host targeting agents (HTAs), such as miRNA inhibitors, is believed not to lead to the same rapid emergence of resistance variants compared to directing acting antivirals (DAAs). HTAs may therefore provide a solution for patients infected with DAA resistant strains [84]. Nonetheless, fit miR-122 independent HCV variants have been observed in culture, which does not exclude that the virus may find alternative mechanisms of resistance and escape [85,86]. Seed site randomization and mutagenesis of miR-122 binding sites on HCV have demonstrated that the virus can evolve to acquire mutations that re-direct its tropism by selecting other available cellular miRNAs or allow it to replicate without binding miRNAs [76,87]. Clinical studies identified the presence of putative resistance variants in connection with miR-122 antagonist therapy; in vitro studies supported the resistance associated phenotype [88].

The AGO-CLIP methodology allows identification of miRNA interaction sites on target RNA and represents a critical step towards a more comprehensive understanding of miRNA regulation [89,90]. This has been reviewed in detail [91], including by Gay et al. in this issue [92]. Improvements of the AGO-CLIP method by proximity ligation of miRNA-target chimeras further enable identification of the specific miRNA responsible for AGO binding [93,94,95]. This advance allowed discovery of other virus-miRNA interactions in a study of a broad panel of RNA viruses [57]. Of particular significance was the interaction between the 3′ UTR of bovine viral diarrhea virus (BVDV) and the cellular miRNA families miR-17 (incl. miR-17, -20, -93, and -106) and let-7. BVDV is a (+)ssRNA virus and a member of the *Pestivirus* genus, an important group of animal pathogens (also containing classical swine fever virus, CSFV) distantly related to HCV within the *Flaviviridae* family. Like HCV, the BVDV genome contains highly structured, non-capped, non-polyadenylated UTRs and a 5′ UTR IRES. An RNA structure without 5′ cap and 3′ poly-A tail may indeed be necessary to avoid canonical miRNA repression [96]. Similarly to the HCV/miR-122 interaction, impairing the interaction between miR-17 and BVDV with antisense oligos or site-directed mutagenesis strongly attenuated viral replication. Despite binding to the 3′ UTR, miR-17 also stimulated IRES-dependent viral translation and played a role in protecting the viral genome from RNA decay. The let-7 and miR-17 binding sites are highly conserved across all sequenced pestiviruses supporting a conserved function within this genus. Mutating the miR-17 seed site of CSFV or inhibiting miR-17 by antagonists recapitulated the attenuated phenotype although miRNA dependence appeared less absolute than for BVDV. It is thus plausible that other pestiviruses would also depend on miRNAs. Other RNA viruses of the AGO-CLIP based screen displayed more pervasive AGO/miRNA binding with several peaks across the viral genome [57]. Some, such as the *Togaviridae* family members SINV and CHIKV, replicated to high enough levels to exert a general occupancy of AGO/miRNA complexes but with less miRNA specificity. The functional relevance of most other identified interactions remains untested and more interactions of interest may therefore await discovery.

Another recent study found that miR-21 directly interacts with the ZIKV 5′ UTR [97]. Interestingly, ZIKV replication was attenuated in miR-21 knock-out cells, after treatment with miR-21 inhibitors or after mutagenesis of the miRNA binding site. Although further mechanistic studies are warranted, it appears that miR-21 binding may function as a switch to control whether the 5′ and 3′ ends of the viral genome can base pair and thereby circularize the genome. While miR-122 binding to HCV is absolutely critical, the reported impact of miR-21 binding was only 2-fold on viral RNA accumulation, and its biological importance therefore needs to be confirmed.

#### 2.1.3. Viral Modulation of Host miRNA Abundance

Cellular competing endogenous RNAs (ceRNAs) bind miRNAs to regulate specific miRNA pools rather than being regulated themselves [98]. An interesting question, in particular in the case of persistent RNA viruses, is therefore whether viral occupancy of specific miRNAs could affect the available pool of these cellular miRNAs, and by extension the regulation of their natural mRNA targets. Among DNA viruses, herpesvirus saimiri (HVS) downregulates miR-27 by expressing HVS U-rich RNAs (HSURs). These ncRNAs can bind miR-27 to destabilize it by not completely understood mechanisms [99]. Furthermore, infection with human and murine cytomegalovirus (CMV) leads to destabilization of specific miRNAs of the miR-17 and miR-27 families, respectively [100,101]. Whereas the functional impact of ceRNA mediated miRNA sequestration has been debated [102,103], viruses may be a special case since their replication could benefit from miRNA binding thereby providing a positive feedback loop. 

Using AGO-CLIP, it was demonstrated that HCV can sequester enough miR-122 to redirect the miRNA repression away from its endogenous mRNA targets, thereby showing the first such miRNA “sponge” for an RNA virus (Figure 1C) [87]. Using fluorescent single-cell reporters and quantitative modeling as well as miRNA-target chimeras it was estimated that HCV can sponge 40–50% of the available miR-122 pool in cell culture [57,87]. Notably, miR-122 is a tumor-suppressor and knockout mice spontaneously develop hepatocellular carcinoma (HCC) [104,105]. Since HCV infection is causative of around 25% of the overall cases of HCC [106], it is tempting to speculate that the partial virus induced miR-122 sequestration over years of chronic infection provides an environment fertile for liver cancer. Although the causative link is difficult to study in patients, recently developed rat and mouse models for HCV could possibly enable such studies [78,79]. A similar sponge activity was reported for the pestiviruses BVDV and CSFV and the miR-17 family [57]. BVDV genomes harboring a miR-17 binding site were able to efficiently de-repress miR-17 cellular target mRNAs in cell culture, as were CSFV upon infection of porcine macrophages ex vivo. Since miR-17 is a pro-proliferatory miRNA [107], it is interesting to speculate whether pestiviral miR-17 sequestration could provide an indirect, non-immunogenic way of manipulating the immune system by decreasing the available pool of miR-17 in infected antigen presenting cells, thereby limiting immune responses towards the virus. Despite also binding let-7, both pestiviruses failed to functionally sequester this highly abundant miRNA, suggesting that the relative abundance of a miRNA determines any de-repression. Thus, RNA viruses also employ mechanisms of direct RNA binding to regulate the functional abundance of specific cellular miRNAs.

### 2.2. Other RNAs

With several cases of miRNAs interacting with viral RNA, interactions with other types of cellular RNA would be unsurprising. Systematic identification of such has only recently become possible through methods like RNA antisense precipitation (RAP) where the RNA of interest is pulled down by specific probes after RNA–RNA cross-linking using psoralen derivatives and UV_365_ [108], as well as global capture methods allowing identification of all cross-linked RNAs after proximity ligation [109,110,111]. An example from the world of DNA viruses is that of HVS HSUR2. Application of RNA pull-down and sequencing methodology identified that HSUR2 can bridge miR-16 and miR-142-3p to specific mRNAs by interacting simultaneously with both, resulting in the repression of the mRNA [112]. Only few examples of RNA viruses engaging in direct base-pairing interactions with host RNAs other than miRNAs have been described so far, and these are discussed below.

The *Retroviridae* family is a large group of RNA viruses able to stably integrate a pro-viral form of their genome into the host genome. Before genome integration, the retroviral RNA genome is converted into DNA by the viral reverse transcriptase (RT) [113,114]. To initiate this process, the RT enzyme uses specific host tRNAs as primer; the 3′ end of the host tRNA is engaged in sequence specific interaction with the complementary primer binding site (PBS) on the viral genome (Figure 1D) [113]. Through a series of steps and the use of repeat regions present in both ends of the genome, viral single-stranded RNA is converted to double-stranded DNA. Although all known retroviruses use cellular tRNAs to prime reverse transcription, different genera use different tRNAs (Table 1). Human lentiviruses, such as HIV, use tRNA^Lys^_3_, whereas murine leukemia virus (MuLV), a gammaretrovirus, uses tRNA^Pro^ [61]. In vitro experiments using HIV-1 demonstrated that during short-term culture of a virus with altered PBS unable to bind tRNA^Lys^_3_, spontaneous reversion to wild-type sequence occurs [58]. Moreover, stable HIV-1 PBS mutants binding alternative tRNAs and unable to revert to the wild-type sequence displayed an attenuated replicative phenotype, suggesting that the selection of specific tRNAs may affect viral fitness [58]. Recently, additional sequences embedded in the HIV genome complementary to tRNA^Lys^_3_ have been described to promote the reverse transcription process [59]. It was further shown that the viral nucleocapsid protein (NC) is crucial in facilitating tRNA-PBS interactions and stabilizes reverse transcription intermediates [59]. The NC activity depends on its interaction with the PBS, the 3′ end of the tRNA and additional cis-genomic elements in a sequence specific fashion to liberate complementary sequences of the PBS and tRNA for intermolecular interaction [60]. This may explain why different retrovirus genera encoding specific NC proteins use different tRNAs.

Other examples of direct RNA–RNA interactions of functional importance between RNA viruses and host cell RNA are currently lacking. A recent study employing a combination of RAP and proximity ligation on ZIKV infected cells identified direct interactions with miRNAs, tRNAs, and U1 snRNA [97]. However, no functional experiments were done except for on the interaction with miR-21 (see above). It will thus be interesting in the future to follow whether methodological advances allow identification of interactions between RNA viruses and other types of cellular RNA molecules.

## 3. Indirect Interactions of RNA Viruses and Host RNAs

A large number of host regulatory RNAs, in particular miRNAs, are important during infection. These RNAs are regulated or have changed localization upon viral infection or are important for the modulation of viral host factors or immune responses upon viral infection. Their regulation therefore contributes indirect effects on infection, although many such effects may occur as secondary effects of infection with no direct pro- or anti-viral role. Whereas it is beyond the scope of this review to comprehensively cover all such indirect regulations, we discuss below selected examples with functional pro- or anti-viral effects. Such modulation could regulate antiviral responses, immune system activation or promote viral replication and further may depend on tissue context. Other recent reviews more comprehensively cover indirect virus-miRNA [115,116] and virus-lncRNA interactions [117].

### 3.1. Micro-RNAs

Above, we discussed the case of EEEV and miR-142-3p, demonstrating how specific miRNAs can restrict cell tropism of RNA viruses via direct interaction. Physiological levels of miRNA subsets also appear to be critical in determining the degree of permissiveness to infection of several neurotropic RNA viruses without direct targeting the viral genome. miR-132, a repressor of the histone acetyltransferase complex p300/CBP, is known to decrease the expression of IFN stimulated genes (ISGs). Interestingly, higher permissiveness of cortical neurons compared to granule cell neurons positively correlated with higher miR-132 expression, thus potentially increasing permissiveness in specific brain areas for positive-stranded RNA viruses such as Saint Louis encephalitis virus (SLEV), WNV, and VEEV [118]. 

**Figure 2:**
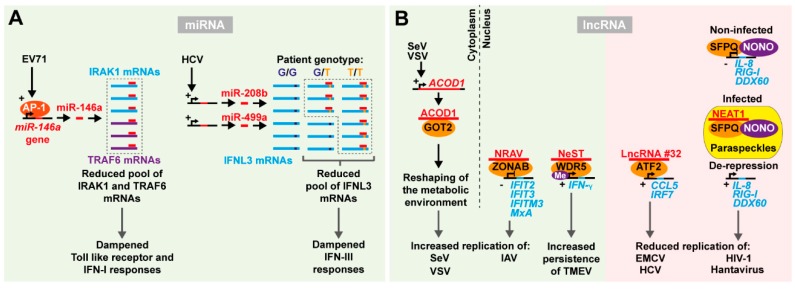
Indirect interactions between RNA viruses and host ncRNAs affect viral replication. (**A**) MiRNA expression induced by viruses to dampen IFN responses. When cells are infected by EV71, miR-146a expression is induced by the transcription complex AP-1. MiR-146a then binds and represses its mRNA targets, IRAK1 and TRAF6, impairing the activation of Toll-like receptors and the IFN-I response. HCV infection upregulates the miR-208b/499a cluster. These miRNAs target the 3′ UTR of IFNL3 mRNAs, counteracting the activation of the IFN-III response. In patients carrying a specific single nucleotide polymorphism in the miRNA seed region (G (dark blue) instead of T (orange)) targeting is abolished resulting in restored gene expression. This polymorphism is associated with clearance of the infection versus persistence. (**B**) LncRNAs affect viral infection. LncRNA ACOD1 accumulates in the cytoplasm upon SeV and VSV infection and binds the GOT2 protein to re-shape the metabolic landscape of the cell and promote viral replication. In the nucleus, different mechanisms with opposite effects can take place: lncRNA NRAV binds the transcription factor ZONAB to repress ISGs and promote IAV replication; lncRNA NeST binds the histone methyl transferase WRD5 leading to histone methylation and expression of the *IFNG* gene, which increases TMEV persistence; on the contrary, binding of lncRNA #32 to the transcription factor ATF2 activates ISGs and reduces replication of EMCV and HCV; a similar effect is observed for HIV-1 and hantavirus due to the derepression of ISGs following the sequestration of the transcription regulator complex SFPQ-NONO by lncRNA NEAT1 to paraspeckles.

Several studies suggest that RNA viruses induce the expression of cellular miRNAs targeting regulators of IFN signaling. One example is that of the HCV-induced miRNAs, miR-208b and miR-499a targeting the type III IFN lambda. Single nucleotide polymorphisms in the region encoding IFN lambda 3 (*IFNL3*) and 4 (*IFNL4*) are associated to acute or chronic outcome of HCV infection [119]. Interestingly, miR-208b and miR-499a target only specific variants of the *IFNL3* 3′ UTR thereby contributing to decay of the IFNL3 genotype associated with chronicity but not the variant associated with clearance [120]. The same two HCV-induced miRNAs were subsequently shown to also dampen type I IFN signaling through repression of the IFN-α receptor 1 (IFNAR1) [121]. These data collectively suggest that HCV counteracts the cellular innate immune response coordinating the expression of miRNAs, intersecting and dampening both the type I and type III IFN pathways (Figure 2A).

EV71 infection upregulates miR-146a levels through the activation of the AP-1 transcription complex [122]. High levels of miR-146a are responsible for the post-transcriptional repression of IRAK1 and TRAF6, involved in the Toll-like receptor (TLR) signaling and type I IFN production (Figure 2A). Interestingly miR-146a knockout mice survived EV71 infection longer than wild type mice, displayed a higher expression of IRAK1 and TRAF6 and sustained IFN production. In addition, LNA antagomir based repression of miR-146a levels in wild type mice increased the expression of type I IFN and ISGs upon infection, limiting viral spread and increasing survival [122]. These data suggested that targeting host miRNAs induced by viral infection may provide valid alternatives in designing antiviral therapies.

### 3.2. Long Non-Coding RNAs

Sequence analysis of the mammalian transcriptome uncovered a vast transcriptional landscape of regulatory lncRNAs both overlapping (sense or anti-sense) and outside known protein coding and small RNA genes [25,123,124]. Depending on their sub-cellular localization, lncRNAs can modulate metabolism and gene expression by coordinating architecture of nuclear domains, mediate epigenetic modifications, and regulate translation and stability of target mRNAs and protein functions [124,125]. Although less conserved than protein coding genes [126], the expression of specific lncRNAs, such as XIST or FENDRR, is essential for embryonic development and organ homeostasis [127,128,129].

Specific lncRNA expression signatures can be induced as part of the innate immune response to viral infection. Infection with the (+)ssRNA virus, SARS-CoV, promotes differential expression of over a thousand lncRNAs in infected mice [130]. The extent of specific lncRNA expression correlated with activation of the IFN response. Infection with IAV recapitulated this signature, suggesting a general mechanism of lncRNA expression involved in the regulation of the antiviral response [130] (Figure 2B). The first functional evidence for a lncRNA in the antiviral response was the identification of NeST [131]. This lncRNA is conserved and expressed from the same locus but on the opposite strand to the *IL22* and *IFNG* genes. CD8+ T-cells from transgenic mice over-expressing NeST are more susceptible to persistent infection by the murine (+)ssRNA virus, Theiler’s murine encephalomyelitis virus (TMEV) of the *Picornaviridae* family. WDR5, a histone methyl transferase, interacts with NeST to alter histone methylation of the *IFNG* promoter, thereby stimulating its expression. Despite many lncRNAs epigenetically regulating neighboring genes, NeST apparently works in trans also when expressed from distant genomic loci. Curiously, NeST expression decreased susceptibility to bacterial (*Salmonella*) infection, whereas it increased TMEV persistence. The authors speculate that this conundrum may be caused by NeST altering the magnitude or timing of inflammatory responses, activating basal inflammation to attenuate subsequent inflammatory events, or alternatively having other targets in addition to IFN-γ.

Replication of IAV in human cells is stimulated by the expression of the lncRNA negative regulator of antiviral response (NRAV) [132], the expression of which is markedly reduced upon IAV infection. Similar behavior was observed upon infection with different species of RNA and DNA viruses, but not other pathogens, suggesting that NRAV repression is a general mechanism of the cellular antiviral defense. Manipulating NRAV expression levels demonstrated that repression of this lncRNA is required to sustain an efficient antiviral response and rapidly clear IAV infection. Several ISGs appeared to be repressed by NRAV, including IFIT2, IFIT3, IFITM3, and MxA. Moreover, transgenic mice over-expressing NRAV rapidly succumb to IAV infection. As for NeST, NRAV function is also linked to its ability to regulate epigenetic modifications. NRAV recruits the transcription factor ZO-1-associated nucleic acid binding protein (ZONAB) to the promoters of ISGs leading to ISG repression. On the contrary, lncRNA #32 is positively correlated with type I IFN signaling and stimulation of ISG production [133]. LncRNA #32 repression increased susceptibility to infection with the (+)ssRNA encephalomyocarditis virus (EMCV) and impaired IFN responses against HBV and HCV in primary cells. The expression of several ISGs and chemokines, including CCL5 and IRF7, was significantly repressed in lncRNA #32 depleted cells. RNA pulldown using biotinylated lncRNA #32 identified the ISG associated transcription factor, ATF2. The suppression of lncRNA #32 by IFN-β in a feed-back loop may protect the cell from excess inflammation caused by high ISG expression. These data suggest that lncRNAs may specifically affect the transcriptional activation of ISGs by directly recruiting transcription factors or epigenetic regulators to target promoter sequences thereby influencing antiviral responses. 

The expression of certain nuclear lncRNAs coordinates the assembly of functional nuclear domains, or nuclear bodies. These highly specialized compartments contain molecules involved in regulation of gene expression, including splicing and transcriptional activation. The NEAT1 lncRNA is a main constituent of nuclear paraspeckles and its expression is essential for paraspeckle formation and function. NEAT1 expression is induced upon infection with several RNA and DNA viruses, leading to a rapid accumulation of paraspeckles in the nucleus of the infected cells [134,135,136]. NEAT1-dependent paraspeckle formation induces the re-location of specific transcription factors away from other nuclear domains. The splicing factor proline- and glutamine-rich protein (SFPQ) is a nuclear RNA binding protein that among other functions inhibits gene expression at the transcriptional level in presence of the non-POU domain-containing, octamer binding protein (NONO). NEAT1 is known to directly bind SFPQ and thereby remove it from its target promoter and relocate it to the paraspeckles. A number of important ISGs, such as RIG-I, DDX60 and IL-8, are repressed by the SFPQ/NONO complex in absence of viral infection. Upon infection, e.g., with HIV-1 or hantavirus, a (−)ssRNA emerging hemorrhagic fever related virus, this repressor complex is relocated to the paraspeckles through NEAT1 interaction, alleviating repression of ISGs, that then counteract the viral infection [134,135,136].

RNA viruses have also evolved mechanisms to manipulate and hijack host lncRNAs to promote viral replication [137]. lncRNA-ACOD1 is induced by multiple viruses, including the (-)ssRNA Sendai virus (SeV) and VSV in multiple tissues. Interestingly, lncRNA-ACOD1 repression in cultured cells as well as deletion in lncRNA-ACOD1 knockout mice is sufficient to dramatically reduce viral replication independent of IFN responses. RNA pulldown experiments elucidated that lncRNA-ACOD1 function in viral replication depends on its interaction with a protein factor, the glutamic-oxaloacetic transaminase 2 (GOT2), critical for the amino acid glutamine metabolism. The authors proposed a model in which allosteric conformational change induced by the interaction of the lncRNA in proximity of the substrate site of the enzyme would enhance GOT2 activity. This would reshape the cellular metabolic environment and mobilize energy that the virus can exploit for replication and particle production. Interestingly, in this case such a mechanism takes place in the cytoplasm while other examples described above occur in the nucleus through transcriptional regulation.

Thus, several types of cellular ncRNA, including miRNAs and lncRNAs, are regulated upon viral infection—as a cellular response to infection or as a viral evasion strategy—and the outcome can be either pro- or anti-viral. Many more such cases of miRNAs and lncRNAs may await discovery, and future studies will show whether other regulatory RNA molecules exhibit similar responses to infection.

## 4. RNA Virus Encoded Non-Coding RNA

In addition to viral exploitation of cellular RNA, virus derived ncRNA play important functions. For large DNA viruses like herpesviruses, expression of several classes, including miRNAs and lncRNAs, is well characterized [40,138]. Here we discuss current insights on ncRNA species derived from RNA viruses.

### 4.1. Function of Virus Derived Micro-RNAs

Despite a number of DNA viruses producing virus derived miRNAs, we currently lack robust evidence of true RNA viruses producing functional miRNAs [138,139]. MiRNAs derived from the 3′ UTRs of WNV and DENV were, however, described [140,141]. The WNV miRNA is produced by miRNA processing machinery in mosquito cells and appears to be functional on reporter assays. It is suggested to be important for viral replication in insect cells [140], however, the reliance on miRNA inhibitors only makes interpretation difficult as those would also be expected to directly target the viral genome and potentially inhibit stem-loop formation. The production of biologically functional DENV derived miRNAs has been questioned given their markedly low abundance, RNAi like biogenesis and lack of conservation [142,143]. Explanations for lack of RNA virus encoded functional miRNAs could include that most RNA viruses replicate in the cytoplasm, preventing access to miRNA biogenic machinery. However, cytoplasmic translocation of Drosha was demonstrated during infection [144], and RNA viruses with engineered miRNA expression could be processed directly by Dicer [145,146]. Furthermore, RNA viruses containing miRNA hairpins would have their genome cleaved directly during miRNA processing, whereas their anti-genome would be a cleavage target for RNA interference (RNAi). This would presumably result in significant attenuation of viral replication. Nonetheless, by incomplete processing or compartmentalization, these constraints could still be compatible with viral replication. Although the fine-tuning of gene expression exerted by miRNAs may be irrelevant during the rapid acute infection of most RNA viruses, it could still be imagined that persistent RNA viruses, such as hepaci-, pegi-, and pestiviruses, could find use for expressing virus derived miRNAs.

Viral miRNA expression has indeed been demonstrated for the retroviruses, simian foamy virus (SFV), and bovine leukemia virus (BLV). Given their DNA intermediate, retroviruses would not necessarily experience the restrictions discussed above. BLV and SFV contain miRNA encoding sequence clusters overlapping introns and in the long terminal repeat sequence of the viral genome, respectively. Whereas most canonical primary miRNA transcripts are transcribed by RNA pol II and processed by the Drosha complex into pre-miRNAs, the SFV and BLV miRNA primary transcripts are produced by RNA pol III and processed to mature miRNAs through a Dicer-dependent Drosha-independent mechanism [147,148]. Some of these viral miRNAs are functionally loaded on the RISC complex and carry out post-transcriptional repression of host transcripts. SFV encoded miR-S4-3p, for example, mimics the seed sequence of cellular miR-155, while miR-S6-3p mimics miR-132. Several targets of miR-155 regulate cell proliferation, leading to the hypothesis that viral miRNAs, such as SFV-miR-S4-3p, stimulate proliferative activity of SFV infected cells [148]. Similarly, miR-S6-3p was shown to be a functional mimic of the IFN-suppressive miR-132, thereby helping the virus escape innate immunity. BLV-miR-B4-3p mimics the seed sequence of miR-29, a miRNA known to be over-expressed in a variety of lymphoproliferative disorders, also suggesting that viral miRNA expression may sustain proliferation of the infected cells and play a role in BLV associated tumorigenesis [147]. In addition, HIV encoded miRNAs were reported, but their low abundance, lack of detection using AGO-CLIP [65], and lack of evolutionary conservation and biological role question their functional relevance [149].

### 4.2. Viral Derived Small Interfering RNAs as Part of the RNA Interference Response 

Deep-sequencing of virus-infected cells showed accumulation of a variety of viral-derived RNA species. In several eukaryotic organisms, sensing of viral replication intermediates triggers the activation of the antiviral RNAi. In many organisms, orthologues of Dicer are able to detect and ‘dice’ foreign dsRNA into ~21–22 nucleotides small interfering RNAs (siRNAs) that guide AGO slicing activity. This mechanism works in parallel to the miRNA machinery, albeit resulting in the cleavage of the RNA target. Given the presence of dsRNA intermediates during RNA virus replication, this mechanism targets the viral genome [149,150,151]. Antiviral RNAi mechanisms were extensively dissected in plants and invertebrates [152,153,154]; however, whether a similar antiviral siRNA signature is observed and functional in infected mammalian cells has been debated [155]. To clear viral infections, mammals have evolved a robust innate immune system based on the interferon system. Nevertheless, mammalian cells still conserved the RNAi pathway including components, such as Dicer and AGO2 proteins, capable of mediating viral RNA cleavage. Indeed, in mouse pluripotent stem cells where the interferon system is attenuated, viral derived siRNAs could be detected upon EMCV infection [156]. This siRNA production was abrogated in Dicer deficient cells. Interestingly, differentiating RNAi competent stem cells into somatic cells resulted in attenuation of small RNA production, probably due to an increase in interferon related gene expression; the depletion of Dicer or AGO2 in somatic cells had no significant impact on viral replication. These observations supported the idea that antiviral RNAi generally does not play a major role in mammalian somatic cells, or that any effect would be limited to progenitor cells [157]. Despite this, a parallel study did observe evidence of active RNAi in BHK cells [158]. Subsequent studies, however, delineated that RNAi is functional in somatic cells, and that this RNA-based mechanism may serve as an underlying innate defense when the protein-based interferon system is not active [159].

Recently, several studies suggested that the difficulty in detecting antiviral RNAi activity in somatic mammalian cells may be due to mammalian specific RNA viruses encoding viral suppressor of RNAi (VSR) proteins. The insect specific (+)ssRNA nodamura virus (NoV) B2 protein is a classic example of a VSR, depletion of which induces siRNA accumulation in NoV infected cells. Interestingly, this can be rescued by heterologous proteins with VSR activity, including the EBOV VP35 protein [158,160]. Similarly, IAV NS1 inhibits viral derived siRNA accumulation in human cells and an IAV NS1 deletion mutant was highly attenuated in AGO2 expressing cells [161]. However, IAV NS1 is also a potent inhibitor of interferon signaling which makes it difficult to distinguish between its impact on the interferon and the RNAi pathways. A recent screen for viral proteins with VSR activity further identified the ability of the EV71 3A protein to impair Dicer-dependent siRNA production in human and murine cells [162]. EV71 3A counteracts the sensing of foreign RNA by Dicer, which allows the accumulation of viral dsRNA replication intermediates in the cytoplasm and therefore promotes viral replication. Finally, VSR activity was also suggested for the YFV capsid protein [163]. Considering that interferon deficient mammalian cells still may retain RNAi activity, and that many mammalian viruses express proteins inhibiting the innate immune system, it is conceivable that mammalian specific viruses may have a need to employ VSR-like mechanisms to counteract RNAi. While the above examples highlight this, the extent of VSR activity encoded by known mammalian RNA viruses is unknown. It is therefore possible that mammalian RNA virus infections may result in viral derived siRNAs, at least under conditions where RNAi is active and not sufficiently repressed by VSR activities.

### 4.3. Flaviviral Subgenomic Non-Coding RNAs 

**Figure 3:**
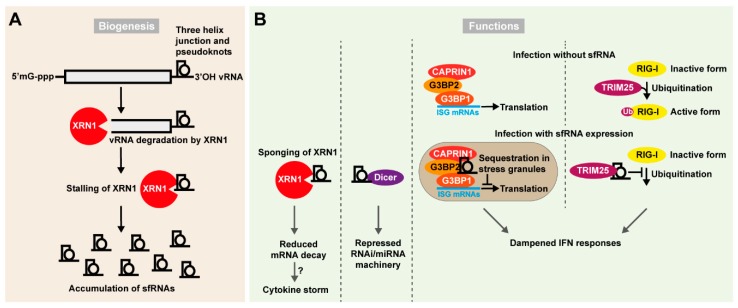
Sub-genomic flavivirus RNAs (sfRNAs) impair cellular response to viral infection. (**A**) Biogenesis of sfRNAs. A pseudoknot-stabilized three junction helix is formed in the 3′ UTR of flaviviral genomes. This blocks the 5′ to 3′ processing and nucleolytic activity of the exonuclease XRN1, which triggers accumulation of sfRNAs in the cell. (**B**) Functions of sfRNAs. The accumulation of sfRNAs impacts several cellular pathways which can favor viral infection: in one mechanism, stalling at the sfRNA pseudoknots sequesters XRN1 away from its usual targets decreasing mRNA decay, including decay of transcripts involved in the antiviral response. This further could contribute to the cytokine storm observed upon infection. In another mechanism a high amount of sfRNAs saturates Dicer and consequently inhibits the RNAi/miRNA pathway. sfRNAs have also been reported to accumulate in stress granules and interact with CAPRIN1, G3BP2, and G3BP1 that are important for translation of ISG mRNAs. Finally, sfRNAs may interfere directly with the PRR mediated IFN-I activation pathway. RIG-I is a central component of PRR signaling and is activated by ubiquitination via TRIM25 upon infection. sfRNAs have been shown to directly bind TRIM25 and by doing so preventing the ubiquitination of RIG-I. This in turn would lead to a dampened IFN response.

The arthropod-borne flaviviruses is a large group of (+)RNA viruses, including WNV, DENV, YFV, ZIKV, and tick-borne encephalitis virus (TBEV), responsible for millions of infections worldwide every year. The capped genome is organized with 5′ and 3′ UTRs flanking a single open reading frame and is not polyadenylated. Mutations in highly structured regions of the viral 3′ UTR significantly compromise the replication of flaviviruses. Structural analysis of a particular region of the WNV Kunjin strain, identified the presence of specific stem-loop (SL) structures necessary for the production of ~0.3–0.5 kb non-coding sfRNAs (Figure 3A) [164]. Interestingly, sfRNA is of great importance to flaviviruses, as viruses specifically defective for its production display a highly attenuated phenotype [164,165,166,167,168,169,170,171]. The sfRNAs are produced through incomplete degradation of the viral genome once engaged by the XRN1 exonuclease, a major component of the 5′-3′ mRNA decay pathway [168,169,170,171]. Three-helix junctions stabilized by pseudoknots form these exonuclease inaccessible XRN1-resistant RNA sequences (xrRNAs). Interfering with xrRNA structures abrogates sfRNA accumulation in infected cells. Thanks to crystallographic resolution of the interaction interface between XRN1 and specific sequences in the DENV and ZIKV 3′ UTRs, mechanistic details of the sfRNA biogenic process are now becoming clearer. While engaged on the target viral genome, the XRN1 exonuclease is stalled on xrRNAs containing single (or multiple) pseudoknot-stabilized tertiary structures, from where it is unable to further degrade the viral genome [169,170]. Two different but conserved architectures of xrRNAs have been identified, correlating with flavivirus phylogeny [171]. Furthermore, the number of different sfRNAs produced differ between flaviviruses. Mutations that stabilize such pseudoknots may trigger an increase in sfRNA accumulation and possibly also in the epidemic potential of the virus [169,170].

The biological functions of sfRNAs vary between organisms infected (Figure 3B). WNV derived sfRNAs in the mosquito *Culex pipiens* accumulate in midgut cells and display RNAi suppressor activity by counteracting Dicer. This process modulates transmission of the virus to the salivary glands and thereby its transmission to the next host [172,173]. In mammalian somatic cells, sfRNAs have been shown to globally inhibit inflammatory gene expression and dampen the cellular type I IFN response interfering with activation of pattern recognition receptors (PRRs) [166,167,174]. Cells can rapidly modulate global gene expression in response to stress through induction of the mRNA decay pathway. Therefore, XRN1 ”sponging” at viral pseudoknots can result in global increase in the stability of mRNA targets otherwise targeted by this exonuclease [167]. It has been hypothesized that the cytokine storm associated with some flavivirus infections may result from increased stability of cytokine mRNAs otherwise targeted from degradation. Furthermore, sfRNAs may be able to inhibit ISG translation through binding of G3BP1, G3BP2, and CAPRIN1 [175]. In addition, sfRNAs were described to impair the innate immune response by direct targeting of cytoplasmic PRRs. Infection of wild-type and interferon-defective IRF3^-/-^-IRF7^-/-^ or IFNAR^-/-^ mice with sfRNA defective WNV strains demonstrated that sfRNA production is critical to sustain infection in animals with intact interferon response [166]. An alternative mechanism of sfRNA blunting of innate immunity was shown by a study identifying a DENV strain accumulating particularly high levels of sfRNAs, which would directly bind the E3 ubiquitin ligase TRIM25 in a sequence specific fashion [174]. TRIM25 is central in promoting ubiquitination of RIG-I, an important PRR recognizing 5′ tri-phosphorylated RNA. The sfRNA inhibition of TRIM25 thus impairs the ubiquitination of RIG-I and thereby its activation. This study further linked the high accumulation of sfRNA of particular DENV-2 strains to their epidemic potential [174]. Thus, some RNA viruses appear to have found ways to functionalize RNA degradation products and take advantage of these as important virus derived ncRNAs with several suggested biological functions.

### 4.4. Retroviral Long Non-Coding RNAs

Whereas viral lncRNAs are well described for large DNA viruses like the PAN RNA expressed by Kaposi’s sarcoma-associated herpesvirus (KSHV) [40], lncRNAs are typically not produced by RNA viruses. For HIV, however, lncRNA expression from the LTR anti-sense to viral gene expression was described [176]. The authors suggested that this lncRNA suppresses viral gene expression, e.g., to regulate latency, and that it may be involved in epigenetic regulation of HIV. However, further studies are needed to address the robustness of this mechanism.

## 5. Final Remarks

In recent years, there has been an increasing interest in developing tools to systematically dissect the biological significance of RNA interactions governing cellular processes as well as pathogen–host interactions. These novel methodologies are expanding our knowledge on gene expression regulation mediated by ncRNAs. It is also becoming clear that such mechanisms play central roles in the regulation of the cellular response to viral infection. Direct binding to cellular ncRNAs is further exploited by RNA viruses to their own advantage, and this could provide new modalities for therapy. Thus, virus–host RNA interactions are becoming a novel focus area to fully understand the complexity of the virus life cycle, but more systematic investigation is required. The role of cellular and viral-derived small RNA is further along compared to lncRNAs and other RNA species. Despite such progress, much remains to be investigated to understand which virus–host RNA interactions are the result of the cell attempting to clear the infection and which represent a viral strategy to create a favorable host cell environment. Lastly, the often narrow focus on pathogenic viral species suggests that a broader investigation of the RNA virosphere will reveal exciting discoveries in this emerging field.

**Unanswered questions**

- Are there undiscovered cases of RNA viruses benefiting from miRNA-mediated repression of replication in certain tissues?
- What undiscovered examples of direct viral RNA-miRNA interactions exist and what are their functions?
- Does virus-mediated RNA sequestration have functional consequences for pathology or immune responses?
- Is miRNA manipulation, even for direct use in genome replication, a feature of persistent viruses?
- Does non-coding RNA in post-transcriptional regulation play unappreciated roles during chronic infection?
- What other types of cellular RNA interact directly with viral RNA?
- Do other mammalian RNA viruses accumulate functional non-coding RNA species akin to sfRNAs?

## Figures and Tables

**Table 1:** Direct interactions between viral and cellular RNA with functional impact.

Phenotype	Virus	Family	Cellular RNA	Ref
Repression of replication	EV71	*Picornaviridae*	miR-296-5p	[41]
	PFV-1	*Retroviridae*	miR-32	[42]
	HIV-1	*Retroviridae*	miR-29a	[43]
	VSV	*Rhabdoviridae*	miR-24; miR-93	[44]
Tissue specific repression of replication leading to decreased IFN response	EEEV	*Togaviridae*	miR-142-3p	[45]
Increased RNA replication, translation and stability	HCV, other hepaciviruses	*Flaviviridae*	miR-122	[46,47,48,49,50,51,52,53,54,55,56]
	BVDV, other pestiviruses	*Flaviviridae*	miR-17/20/93/106 family (let-7 family)	[57]
Reverse transcriptase priming	HIV, SIV, FIV, MMTV, others	*Retroviridae*	tRNA^Lys^_3_	[58,59,60]
	HTLV, MuLV, BLV, others	*Retroviridae*	tRNA^Pro^	[61]
	Avian retroviruses	*Retroviridae*	tRNA^Trp^	[61]
